# Disrupted Balance of Gray Matter Volume and Directed Functional Connectivity in Mild Cognitive Impairment and Alzheimer’s Disease

**DOI:** 10.2174/1567205020666230602144659

**Published:** 2023-08-03

**Authors:** Yu Xiong, Chenghui Ye, Ruxin Sun, Ying Chen, Xiaochun Zhong, Jiaqi Zhang, Zhanhua Zhong, Hongda Chen, Min Huang

**Affiliations:** 1 Department of Neurology, the Seventh Affiliated Hospital of Sun Yat-sen University, Shenzhen, Guangdong 518107, China;; 2 Department of Traditional Chinese Medicine, the Seventh Affiliated Hospital of Sun Yat-sen University, Shenzhen, Guangdong 518107, China

**Keywords:** Granger causality density (GCD), volume-based morphometry (VBM), resting-state functional MRI (rs-fMRI), structural MRI, Alzheimer’s disease (AD), mild cognitive impairment (MCI), dementia

## Abstract

**Background:**

Alterations in functional connectivity have been demonstrated in Alzheimer’s disease (AD), an age-progressive neurodegenerative disorder that affects cognitive function; however, directional information flow has never been analyzed.

**Objective:**

This study aimed to determine changes in resting-state directional functional connectivity measured using a novel approach, granger causality density (GCD), in patients with AD, and mild cognitive impairment (MCI) and explore novel neuroimaging biomarkers for cognitive decline detection.

**Methods:**

In this study, structural MRI, resting-state functional magnetic resonance imaging, and neuropsychological data of 48 Alzheimer’s Disease Neuroimaging Initiative participants were analyzed, comprising 16 patients with AD, 16 with MCI, and 16 normal controls. Volume-based morphometry (VBM) and GCD were used to calculate the voxel-based gray matter (GM) volumes and directed functional connectivity of the brain. We made full use of voxel-based between-group comparisons of VBM and GCD values to identify specific regions with significant alterations. In addition, Pearson’s correlation analysis was conducted between directed functional connectivity and several clinical variables. Furthermore, receiver operating characteristic (ROC) analysis related to classification was performed in combination with VBM and GCD.

**Results:**

In patients with cognitive decline, abnormal VBM and GCD (involving inflow and outflow of GCD) were noted in default mode network (DMN)-related areas and the cerebellum. GCD in the DMN midline core system, hippocampus, and cerebellum was closely correlated with the Mini-Mental State Examination and Functional Activities Questionnaire scores. In the ROC analysis combining VBM with GCD, the neuroimaging biomarker in the cerebellum was optimal for the early detection of MCI, whereas the precuneus was the best in predicting cognitive decline progression and AD diagnosis.

**Conclusion:**

Changes in GM volume and directed functional connectivity may reflect the mechanism of cognitive decline. This discovery could improve our understanding of the pathology of AD and MCI and provide available neuroimaging markers for the early detection, progression, and diagnosis of AD and MCI.

## 1 INTRODUCTION

By 2021, Alzheimer's disease (AD) has become the seventh leading cause of death and the most common cause of dementia among people over 65 years of age [1].

The deposition of amyloid-β protein (Aβ) and phosphorylated Tau protein is considered the basic pathological mechanism of cognitive decline, which eventually leads to extensive loss of synapses and neurons [2-4]. However, there are still more than 41 million global cases of dementia without a proper diagnosis [1] because of the lack of effective diagnostic methods. Therefore, research progress in its early detection and treatment has urgent and important significance for cognitive decline.

Volume-based morphometry (VBM) is a recognized method for detecting cognitive impairment, and hippocampal volume reduction has been used as a biomarker for AD [5-7]. However, it is not the most sensitive biomarker because it can also be found in schizophrenia [8] and depression [9, 10]. Hence, studies on functional magnetic resonance imaging (fMRI) were subsequently conducted. In a study by Li *et al.* on the pathophysiology of diseases using resting-state fMRI [11], for the first time, the hippocampus (HIP) was regarded as the area showing a significant difference in analysis between patients with AD and normal controls (NCs). Declining functional connectivity in HIP of AD patients was performed in several studies previously. Subsequently, several researchers put forward a hypothesis about brain networks and confirmed the essential role of the default mode network (DMN) in cognitive decline. According to neuroimaging analysis, Andrews *et al.* [12] first defined DMN as the midline core system and two subsystems (medial temporal lobe (MTL) and dorsomedial prefrontal cortex (dMPFC).

The midline core subsystem of the DMN (mainly involving the precuneus (PCUN), posterior cingulate gyrus, anterior cingulate gyrus (ACG), and middle frontal gyrus (MFG) participates in the assessment of personal meaning or self-relevancy, which is the earliest significant region discovered in cognition impairment. DMN MTL subsystem includes the HIP, parahippocampal gyrus, ventral MPFC, and interior parietal lobe. It mainly constructs memory scenes to match the main manifestations of cognitive impairment. The DMN dorsal MTL (dMPFC) subsystem contains the dorsal MTL, temporoparietal junction, lateral temporal cortex, and temporal pole and functions in self-referential psychological activities. The accepted hypothesis is that DMN activity during rest is necessary for memory consolidation, which indicates that it has a potential connection with AD development [13]. In addition, recent studies have reported that several valuable neuroimaging biomarkers related to DMN are available [14, 15], although the optimal validity of functional connectivity values requires further verification in future studies.

To date, functional neuroimaging has been widely used to study changes in functional networks and relationships among different brain networks in diseases. There are two main approaches to evaluating functional neuroimaging characteristics, namely, functional connectivity (calculating the correlation of time courses between one brain region and others) and functional connectivity density [16] (calculating the functional connectivity strength of the whole brain). However, none of these methods reflect directed information flow among different regions of the brain, which can be reflected through Granger causality analysis based on prior hypotheses of the definition of regions of interest (ROIs). To measure directed functional connectivity, we used a new method called Granger causality density (GCD) [17-19], which aggregates conditional information sets through weighted connectivity density maps to reflect the average connectivity strength between each voxel and the rest of the brain. Furthermore, GCD could provide an unbiased opportunity to search for abnormalities within the entire connectivity matrix without any prior assumptions and reflect the directional information flow among different brain networks from the voxel levels. However, it cannot be ignored that this method has only been applied to epilepsy. In a previous study, due to significant fluctuation in amyloid load, we proved that EEG is a new and useful biomarker of preclinical AD [20], which may enable us to monitor significant changes in directional functional connectivity during cognitive decline. Szabo *et al.* found a bidirectional link between AD and epilepsy, which confirmed the rationality of GCD analysis in patients with mild cognitive impairment (MCI) and AD [13].

This study aimed to determine changes in GCD in patients with AD and MCI compared with those in the control group. To achieve this goal, we first analyzed GCD in the process of cognitive decline and conducted a within-group correlation analysis to disclose the relationship between cognitive function and GCD in the identified abnormal regions. Then, we used VBM as the reference substance of the domain GCD in the structural field and participated in multimodal clinical receiver operating characteristic (ROC) analysis. In the final analysis, we extracted the VBM and GCD values to distinguish among patients with AD and MCI and NCs, with the aim of searching for multimodal neuroimaging biomarkers for the early detection of MCI, cognitive disorder progression and AD diagnosis.

## MATERIALS AND METHODS

### 2.1 Participants

In this study, we took advantage of brain fMRI scans from the Alzheimer’s Disease Neuroimaging Initiative (ADNI; adni.loni.usc.edu), which is available on public websites. The ADNI, founded in 2003, is a public-private partnership project led by Principal Investigator Michael W. Weiner, MD. The primary goal of ADNI has been to test whether serial MRI, positron emission tomography, other biological markers, and clinical and neuropsychological assessments can be combined to measure the progression of MCI and early AD. The up-to-date information can be found at www.adni-info.org. The resting-state fMRI (rs-fMRI) images of a total of 48 participants (16 NCs, 16 patients with MCI, and 16 patients with AD) were obtained from the ADNI1, ADNI2, and ADNI-GO datasets.

### 2.2 Data Acquisition

Patients and NCs from the ADNI datasets underwent 3.0T MRI using the Philips Medical Systems Machine. The rs-fMRI sequences were obtained using conventional echo-planar imaging with field strength = 3.0 T, repetition time (TR) = 3000.0 ms, echo time (TE) = 30.0 ms, flip angle (FA) = 80.0 degrees, slices = 6720.0, spatial resolution = 3.3 × 3.3 × 3.3 mm^3^, and imaging matrix = 64 × 64. More details regarding the MRI protocol can be found on the ADNI website.

### 2.3 Structural Data Prepossessing and VBM Analysis

VBM involves voxel-wise comparison of the local gray matter (GM) volume by analyzing high-resolution T1-weighted MPRAGE images [21]. After RESTplus V1.24 converts the original DICOM scan to the format of the Neuroimaging Information Technology Initiative (http://www.restfmri.net), pre-evaluation of structural data is performed using SPM12 (https://www.fil.ion.ucl.ac.uk/spm/) and Computational Anatomy Toolkit 12 (CAT12), as previously described by Nicastro *et al.* [22]. Briefly, 3D T1-weighted MRI scans were standardized using an affine, followed by non-linear registration to correct the bias field inhomogeneities, and then divided into GM, white matter (WM), and cerebrospinal fluid (CSF) components. The Diffeomorphic Anatomic Registration Through Exponentiated Lie algebra algorithm (DARTEL) was used to normalize the segmented scans into a standard MNI space [23].

### 2.4 Functional Image Preprocessing

Image preprocessing and subsequent processing were performed using customized MATLAB (The Mathworks Inc., Natick, MA, United States) scripts. SPM12 (https://www.fil.ion.ucl.ac.uk/spm/) and RESTplus V1.24 (http://www.restfmri.net) were used to pre-process the rs-fMRI data. The first 10 volumes of each subject were discarded to balance the signal and allow the subject to adapt to scanning noise. The remaining volumes were corrected for the collection time delay between the slices. Head motion correction and spatial normalization were set to standard EPI templates, and the voxel size was resampled to 3 × 3 × 3 mm^3^. Participants with more than a 1.5-mm maximum translation in the x, y, or z directions and/or a 1.5-degree of motion rotation were removed. The rs-fMRI images were then spatially smoothed by convolution of the three-dimensional image using a three-dimensional Gaussian kernel with a full width at half maximum of 8 mm. Linear regression was applied to remove other sources of possible spurious covariates, including Friston’s 24 head motion parameters, overall average signal, WM, and CSF signals. Finally, the time series of each voxel was linearly detrended, and the time band-pass filter was 0.01-0.1 Hz [24].

### 2.5 Voxel-Based GCD Analysis

Details of the GCD method are presented in previous studies. Our calculation was performed by running GCD batch codes in MATLAB, and based on the Granger causality analysis, we optimized the GCD algorithm. Taking the directed graph GCD and limited analyzing conditions into consideration, we calculated two indicators in our study, namely, inflow and outflow connectivity. In short, the GCD algorithm defines the time series of any one voxel of the brain as x and the time series of the remaining voxels as y. Then, the direct linear influence of x on y (F_x−>y_) and that of y on x (F_y−>x_) were calculated voxel-by-voxel across the whole brain. The F_x−>y_ value represents the output information flow from the targeted voxel (x) to whole brain voxels (y), whereas F_y−>x_ represents the input information flow to the targeted voxel (x) from the rest of the brain voxels (y). For the whole brain voxels, a series of F_x−>y_ and F_y−>x_ values were achieved, which have the ability to show the output and input causal effective connectivity, respectively. The density diagram of the output causal influence of the x variable on the y variable was defined by the summation of the F_x−>y_ values (threshold was defined as *p* < 0.05), *i.e.,* outflow connectivity. Similarly, the density map of the influence of the y variable on the input of the x variable (F_y−>x_) was inflow connectivity [17].

### 2.6 Statistical Analyses

SPSS 25.0 (SPSS Inc., Chicago, IL, USA) was used to analyze the demographic and clinical variables. We managed to determine age differences, Mini‐Mental State Examination (MMSE), Geriatric Depression Scale (GDS), Clinical Dementia Rating (CDR), Functional Activities Questionnaire (FAQ), and Neuropsychiatric Inventory Questionnaire (NPI-Q) by analyzing and comparing variance and the gender ratio adopted chi-square (*χ^2^*) test. Statistical significance was set at two-tailed *p* < 0.05. Structural MRI data were analyzed using SPM12 (https://www.fil.ion.ucl.ac.uk/spm/) and Computational Anatomy Toolbox 12 (CAT12). Functional MRI data analysis of covariance (ANOVA) was performed using Seed-based Connectivity Analysis Toolbox (SeeCAT) (https://www.nitrc.org/projects/seecat/), which was used to determine differences between the groups. Subsequently, based on the brain mask from the abnormal brain regions of the ANOVA, the two groups were compared using post-hoc analysis based on a two-sample t-test. We made use of thresholds of two-tailed voxel and cluster-level *p* < 0.05 to determine the significance, which was corrected for multiple comparisons using a Gaussian random field (GRF) [18, 19]. Then, the mean *Z* values of inflow and outflow GCD were extracted from the clusters with abnormal GCD to explore their correlation with clinical cognition function assessment. We calculated the Pearson correlation coefficients between these variables after assessing the normality of the data to determine the strength of the relationship. Finally, we performed ROC analysis using SPSS 25.0 to obtain a sensitive and specific imaging biomarker for predicting the early detection, progression, and diagnosis of MCI and AD.

## 3 RESULTS

### 3.1 Demographic and Clinical Features

Table **1** presents the demographic and clinical characteristics of the AD, MCI, and NC groups. No significant differences were noted among the groups in age (*F* = 0.430, *p* = 0.653), sex (*χ^2^* = 2.170, *p* = 0.338), and GDS (*F* = 3.093, *p* = 0.055) scores. However, significant differences were noted in the MMSE (*F* = 52.875, *p* < 0.001), CDR (*F* =53.289, *p* < 0.001), FAQ (*F* = 54.916, *p* < 0.001), and NPI-Q (*F* = 5.418, *p* = 0.008) scores.

### 3.2 VBM Differences Among Patients with AD, Patients with MCI, and NCs

GM volumes in the bilateral HIP, PCUN, ACG, inferior parietal gyrus (IPL), and left cerebellar crus were significantly different among the groups. Compared with NCs, GM volumes in patients with MCI increased in the left PCUN (PCUN.L), left posterior cingulate gyrus (PCG.L), left MFG (MFG.L), and left cerebellum crus but decreased in the HIP.L, MFG.L, PCUN.R, left ACG (ACG.L), and left angular gyrus (ANG.L). Patients with AD exhibited increased GM volumes in the bilateral PCG, PCUN.L, and right cerebellum crus but decreased GM volumes in the bilateral HIP, MFG, PRUN, PCG, ACG.L, superior temporal gyrus (TPOsup), cerebellum crus, and left IPL (IPL.L). Additionally, in the comparison between patients with AD and those with MCI, GM volumes of the bilateral HIP, PRUN, PCG, and left cerebellum crus decreased, whereas those of the right inferior cerebellum increased (Table **2**).

### 3.3 GCD Metrics Differences among Patients with AD, Patients with MCI, and NCs

**Fig. (1):**
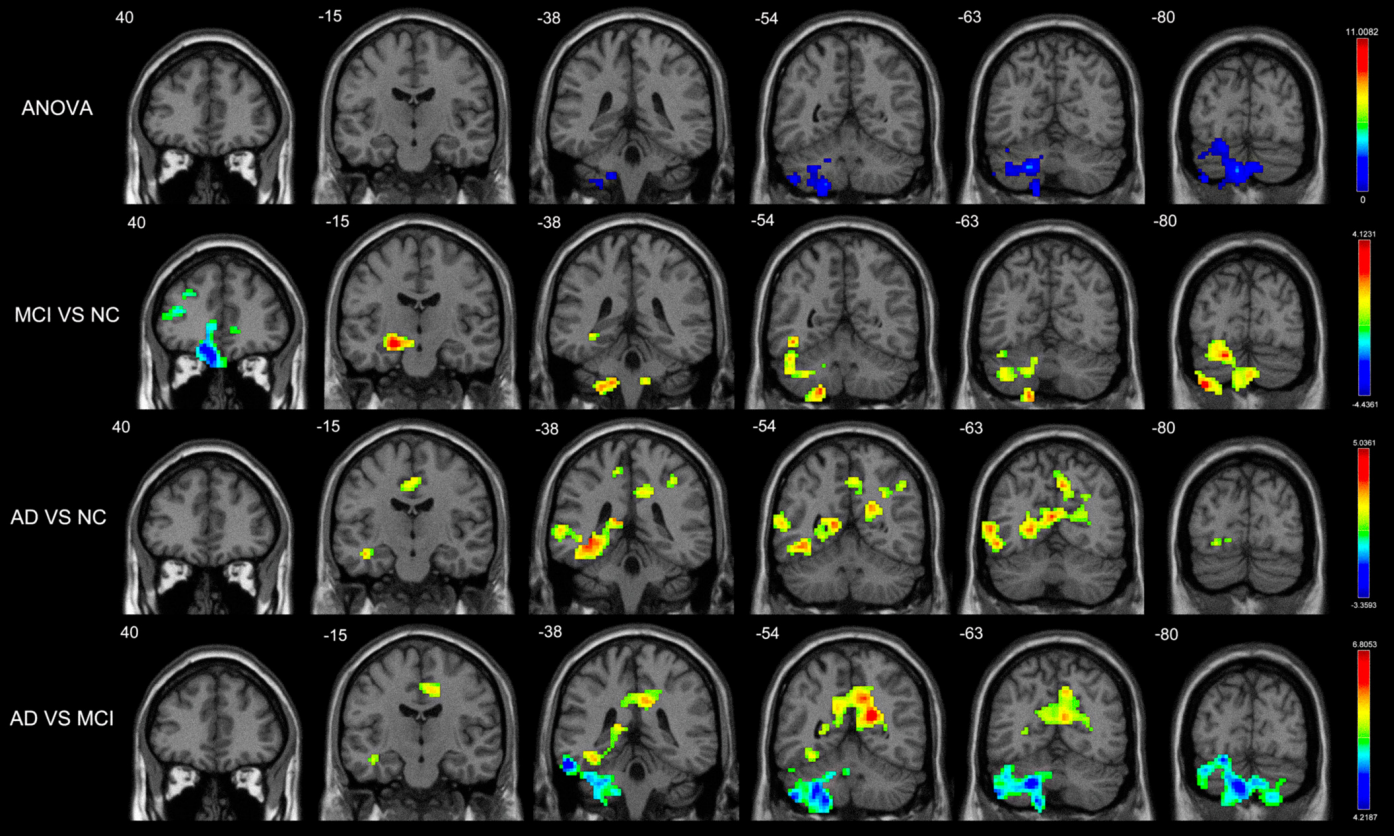
Brain regions with significant differences in inflow GCD (*p* < 0.05, GRF corrected) among Patients with AD, Patients with MCI, and NCs. There were significant among-group differences in inflow GCD in the left cerebellum crus and left inferior cerebellum. Decreased inflow GCD in MCI patients compared to normal controls were located in bilateral ACG and MFG.L while increased inflow GCD in HIP.L, left cerebellum crus and left inferior cerebellum. Increased inflow GCD in AD patients compared to normal controls were located in HIP.L and bilateral PCUN. Decreased inflow GCD in AD patients compared to MCI patients were located in HIP.L, left cerebellum crus and left inferior cerebellum but increased inflow GCD in bilateral PCUN and PCG. **Abbreviations:** ACG, anterior cingulate gyrus; MFG.L, left middle frontal gyrus; HIP.L, left hippocampus; PCUN, precuneus; PCG, posterior cingulate gyrus.

We used the analysis of variance to reveal differences in inflow GCD between the left cerebellum crus and left inferior cerebellum in the cognition decline groups. Based on these results, it can be concluded that inflow GCD was decreased in the bilateral ACG and MFG.L but increased in the left HIP (HIP.L), left cerebellum crus, and left inferior cerebellum in patients with MCI. In patients with AD, inflow GCD was increased in the HIP.L and bilateral PCUN. Further analysis showed that inflow GCD was decreased in the right MFG (MFG.R), bilateral temporal pole in the TPOsup and left cerebellum crus (*p* < 0.01, no correction; Table **S1**). Compared with patients with MCI, patients with AD had a significant difference in the decrease of the inflow GCD in the HIP.L, left cerebellum crus, and left inferior cerebellum but increased inflow GCD in the bilateral PCUN and PCG (Table **3**, Fig. **1**).

**Fig. (2):**
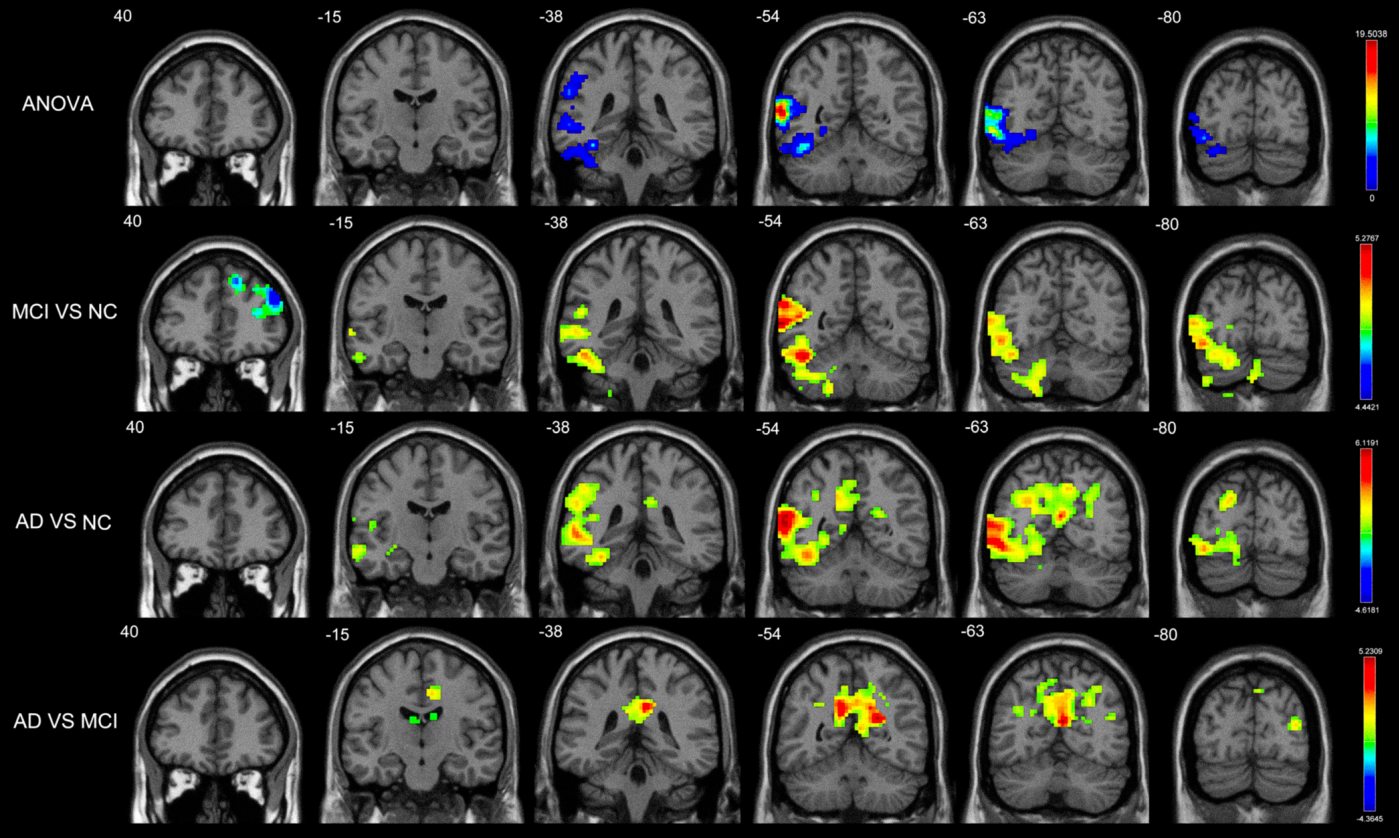
Brain regions with significant differences in outflow GCD (*p* < 0.05, GRF corrected) among Patients with AD, Patients with MCI, and NCs. There were significant among-group differences of outflow GCD in IPL.L and left cerebellum crus. Decreased outflow GCD in MCI patients compared to normal controls were located in MFG.R while increased outflow GCD in ANG.L, left cerebellum crus and left inferior cerebellum. Increased outflow GCD in AD patients compared to normal controls were located in bilateral PCUN and PCG. Increased outflow GCD in AD patients compared to MCI patients were located in bilateral PCG and PCUN.R. **Abbreviations:** IPL.L, left interior parietal gyrus; MFG.R, right middle frontal gyrus; ANG.L, left angular; PCUN, precuneus; PCG, posterior cingulate gyrus.

We found notable differences in outflow GCD in the IPL.L and left cerebellum crus. Compared with NCs, patients with MCI exhibited decreased outflow GCD in the MFG.R but increased outflow GCD in the ANG.L left cerebellum crus and left inferior cerebellum. Patients with AD revealed increased outflow GCD in the bilateral PCUN and PCG. After further analysis, we found that outflow GCD was decreased in the bilateral MFG, TPOsup, and PCUN.R (*p* < 0.01, no correction; Table **S1**). Additionally, outflow GCD increased mainly in the bilateral PCG and PCUN.R in patients with AD compared with patients with MCI (Table **3**, Fig. **2**).

### 3.4 Correlation Between Each GCD Metric and Clinical Cognition Function Assessment

**Fig. (3):**
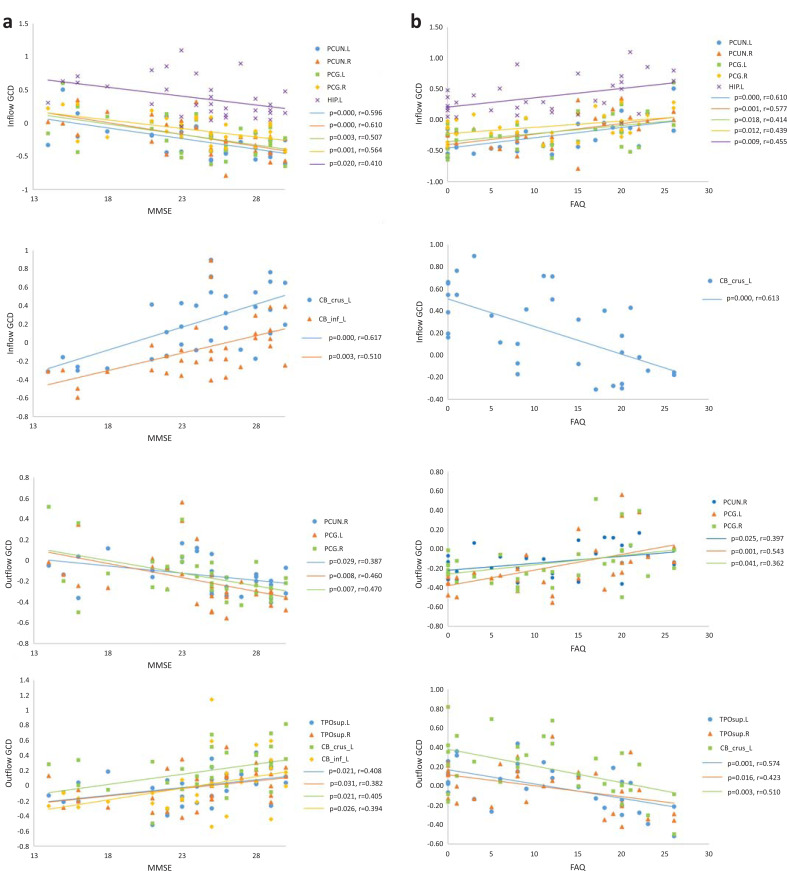
**(a)** Scatterplot of inflow GCD and outflow GCD in specific regions plotted against MMSE score. **(b)** Scatterplot of inflow GCD and outflow GCD in specific regions plotted against FAQ score.

Directional connectivity strength of the inflow and outflow GCD in the brain regions of patients with AD and MCI was strongly correlated with clinical cognitive function assessments (including MMSE and FAQ). Negative correlations were confirmed between inflow GCD and MMSE scores in the regions (including HIP.L, bilateral PCUN, and PCG) related to DMN networks. In contrast, we found positive correlations in the left cerebellum crus and left inferior cerebellum. Correlations between outflow GCD and MMSE scores were negative in PCUN.R and bilateral PCG, whereas a positive correlation was noted in bilateral TPOsup, left cerebellum crus, and left inferior cerebellum (Fig. **3a**).

Positive correlations were found between the FAQ scores and inflow GCD in the regions (including HIP.L, bilateral PCUN, and PCG) related to DMN networks, whereas a negative correlation was noted in the left cerebellum crus. Similarly, positive correlations were noted between the FAQ scores and outflow GCD in PCUN.R and bilateral PCG, whereas negative correlations were noted in bilateral TPOsup and left cerebellum crus (Fig. **3b**).

However, no correlation was noted between the NPI-Q scores and GCD (both inflow and outflow GCD). We did not take GCD and CDR scores into consideration while evaluating the correlation due to the non-significance of GDS scores and the nonlinearity of CDR score data. In addition, GCD values in other brain regions showed no correlation with cognitive function assessments.

### 3.5 Classification Performances of VBM and GCD Metrics

We analyzed the sensitivity and specificity of VBM and GCD in specific regions in the MCI group and NCs, which enabled us to study the effect of early detection of MCI. The area under the curve (AUC) in VBM was the largest in PCG.L, which was 0.750, with a sensitivity of 75.0% and specificity of 75.0%. The maximum AUC of inflow GCD was found in HIP.L, which was 0.797, with a sensitivity of 87.5% and specificity of 68.7%. An equal AUC was noted in MFG.L, with a sensitivity of 87.5% and specificity of 62.5%. In addition, the AUC of outflow GCD was the largest in the left cerebellum crus, which was 0.906, with a sensitivity of 81.3% and specificity of 93.7%. Combined with inflow and outflow GCD, the AUC was the largest in the left cerebellum crus, which was 0.926, with a sensitivity of 93.8% and specificity of 81.2%.

To explore the role of predicting cognitive disorder progression (from MCI to AD), we analyzed the sensitivity and specificity of VBM and GCD in specific regions in the AD and MCI groups. The results showed that in HIP.L, the maximum AUC in VBM was 0.836, with a sensitivity of 81.3% and specificity of 75.0%. The most significant area of inflow GCD was PCUN.R, with an AUC of 0.934, sensitivity of 87.5%, and specificity of 100.0%. In addition, the largest AUC of outflow GCD was in PCG.L, which was 0.918, with a sensitivity of 93.8% and specificity of 81.2%. Combined with inflow and outflow GCD, the largest AUC was found in PCUN.R, which was 0.938, with a sensitivity of 81.3% and specificity of 100.0%.

To explore the effect of clarifying the AD diagnosis, we also analyzed the sensitivity and specificity of VBM and GCD in specific regions in the AD group and NCs. We found that the largest AUC in VBM was noted in HIP.L, which was 0.938, with a sensitivity of 93.8% and specificity of 87.5%. With a sensitivity of 87.5% and specificity of 81.2%, the AUC of inflow GCD was the largest in HIP.L, which was 0.898. Similar to the AUC of 0.898, a sensitivity of 87.5% and specificity of 62.5% were found for the curve of inflow GCD in MFG.L. In addition, the AUC of outflow GCD was the largest in PCUN.R, at 0.836, with a sensitivity of 68.8% and specificity of 87.5%. Finally, combined with inflow and outflow GCD, the most significant region was PCUN.R, with an AUC of 0.887, sensitivity of 81.3%, and specificity of 87.5%.

**Fig. (4):**
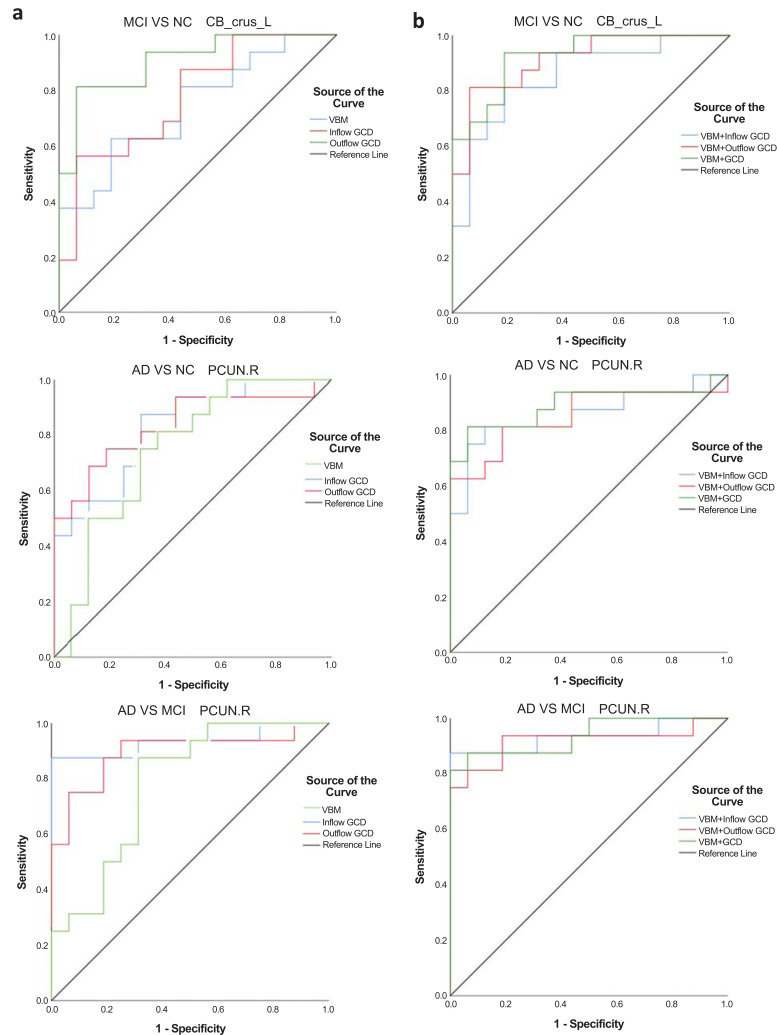
**(a)** Receiver operating characteristic (ROC) curve for left cerebellum crus in the between-group analysis of MCI patients and normal controls, PCUN.R in the between-group analysis of AD patients and normal controls and PCUN.R in the between-group analysis of AD and MCI patients respectively. **(b)** Receiver operating characteristic (ROC) curve for the collaborative effect of VBM and GCD in specific regions to predict MCI early detection and cognitive disorder progression and clarify AD diagnosis.

To clarify the role of VBM combined with GCD in the above three conditions, we conducted further ROC analyses. The left cerebellum crus performed better in the ROC curve, with an AUC of 0.926, a sensitivity of 93.8%, and a specificity of 81.2%, which was superior to the analysis in MCI early detection collaborative prediction. In multimodal cognitive impairment progression prediction, we found the role of PCUN.R, with an AUC of 0.938, a sensitivity of 81.3% and a specificity of 100.0%, which was better than the separated calculation. Regarding AD diagnosis, the AUC was 0.891 in PCUN.R, with a sensitivity of 81.3% and specificity of 93.7%, which was significantly superior to that in the respective analysis (Fig. **4**).

The detailed results of the sensitivity and specificity for the ROC analysis are shown in Table **S2**.

## 4 DISCUSSION

To the best of our knowledge, this is the first study to investigate changes in inflow and outflow GCD in patients with AD and MCI, combined with VBM analysis. This study suggested altered GCD values in DMN-related regions (involving HIP.L, bilateral MFG, PCUN and PCG) and the left cerebellum. GCD values were closely related to clinical impairment (MMSE and FAQ scores) in most regions mentioned above. Furthermore, we found that the combination of VBM and GCD in the cerebellum and PCUN could be used as a biomarker for the early detection of MCI, prediction of cognitive decline progression, and AD diagnosis. These findings may improve our understanding of AD and MCI pathology and promote the development of multimodal neuroimaging biomarkers for practical clinical applications.

In a previous study, EEG was proven to be a new and useful biomarker of the preclinical stage AD due to significant fluctuations related to amyloid burden [20]. Additionally, according to Sperling *et al.* [25], functional connectivity measured by wSMI-alpha in parieto-occipital regions increased due to the local influence of amyloid burden at stage 1 of preclinical AD without neurodegeneration. These findings suggest that changes in directed functional connectivity could be detected and serve as effective biomarkers for the early detection, progression prediction, and diagnosis of cognitive dysfunction. Szabo *et al.* found a bidirectional link between AD and epilepsy [13]. Several studies have also reported on the directed functional connectivity density of epilepsy using GCD [17-19], which indicates that GCD is feasible for functional connectivity analysis of cognitive impairment.

In contrast to other functional connectivity algorithms, the GCD algorithm uses the time series of any one voxel in the brain voxels to calculate the bidirectional linear direct influence with other voxels and the time series of the remaining voxels. The values of inflow and outflow GCD are then added for directed functional connectivity [17]. Ultimately, the principle of GCD algorithm is mainly the relationship between hemodynamic changes and brain neural activity. In the bold series, each voxel contains a time series related to the fluctuations of time-varying blood oxygen levels, which could be related to underlying neuronal activity, oxidative metabolism, neurovascular coupling, and cerebral blood volume [26]. These pathophysiological alterations also manifest in cognition disorders [3, 4], which might enable us to understand the principle that the proposed GCD method has the great potential to distinguish functional bold signals among patients with AD, those with MCI, and NCs. We analyzed two indices of GCD in our study, namely, inflow and outflow GCD. As shown in Table **3**, significant regional differences were noted in inflow and outflow GCD in the AD, MCI, and normal groups. In addition, through GRF correction, the regional performance of these two indicators was found to be similar. Under the same statistical threshold, inflow GCD was more sensitive than outflow GCD in specific brain regions.

In summary, there were three conditions in the among-group comparison combined with VBM and GCD analyses in specific regions. First, both VBM and GCD decreased in DMN midline core system components (involving MFG.L, ACG.L, and PCUN.R), the dMPFC subsystem component (involving bilateral TPOsup), and the cerebellum (mainly involving the left cerebellum crus) in patients with MCI and those with AD. This condition restated the essential role of DMN in cognitive decline, consistent with the findings of previous studies. Amyloid-β deposition and Tau-positive neurofibrillary tangles have been recognized in cognitive impairment pathology [2-4]. The sequence of amyloid-β deposition continuing to rs-fMRI abnormalities, followed by volume loss and finally to cognitive loss, was observed in a previous study [14], which was potential evidence of decreased GM volumes and functional connectivity in our study. As shown by several structural MRI studies, decreased GM volumes in the DMN midline core system indicated comprehensive atrophy of the brain in AD-related to neuron loss [27], which could be connected to amyloid-β deposition and neurofibrillary tangles. In addition, amyloid-β deposition plays a negative role in the functional connectivity of DMN networks. Li *et al.* [28] found that patients with higher cortical amyloid deposition exhibited decreased DMN connectivity, which might lead to worse memory performance. Tau pathology reportedly accumulates early in regions strongly connected to the cortical DMN regions [29] and may have a connection with altered functional connectivity, as reported in a recent work by Ossenkoppele *et al.* [30]. In addition, they found atrophy patterns, which were one of the spatial patterns of Tau pathology across AD phenotypes, and further suggested the spread of Tau *via* network connections. These hypotheses may explain the effect of Tau on the reduction of GM volume and functional connectivity reported in our study.

Second, simultaneously increased VBM and GCD were exhibited in the midline core system components of the DMN (involving bilateral PCG and PCUN.L) and left cerebellum crus in patients with MCI and AD. Barrett *et al.* [31] proposed a hypothesis to explain the resilience of neural damage in the early stages of AD, *i.e.,* optimal compensation of neuron loss. The essential principle is to maintain the excitation/inhibitory balance [32] of injured neurons, which means that if the inhibitory neurons are knocked out, the remaining inhibitory neurons will keep their signal representation error to increase their discharge rates independently of other neurons. Based on this principle, it is reasonable that the DMN midline core system components showed increased GCD in this condition, whereas the adjacent components decreased under the first circumstance. However, neural compensation mostly focuses on vertical compensation, such as increased cerebral blood flow perfusion and metabolism [33], but there are few reports on GM. In recent years, the theory of the compensation ability of GM networks [34] has been proposed, which has confirmed the possibility of GM structure compensation. The results of this study provide a probable mechanism for the increase of VBM in patients with MCI. In addition, in previous studies, GM volumes in patients with cognitive impairment increased, which is a very rare finding. Given our efforts to ensure that our results were reliable and robust, it is unlikely to be a simple false positive. However, we cannot rule out the possibility of aging-induced atrophy and sample heterogeneity in NCs.

In this study, decreased VBM and increased GCD were noted in the DMN midline core system components (involving bilateral PCUN and PCG) and DMN MTL subsystem components (mainly involving HIP.L), which are also DMN network components in patients with AD. Our results are in line with those reported by Busche *et al.* [35], who considered that a decrease in neuronal activity was observed in 29% of layer 2/3 cortical neurons, whereas 21% of neurons displayed an unexpected increase in the frequency of spontaneous Ca^2+^ transients, leading to neuron hyperexcitability. Subsequent studies to explain the hypothesis of neuron hyperexcitability mainly proposed the mechanism of amyloid-β deposition, which may be related to destabilized neural activity in the DMN [36, 37]. Amyloid-β deposition leads to calcium homeostasis, which may result in the degeneration of GABAergic interneurons [38]. Consistent with our findings, Berron *et al.* found that in the presence of amyloid-β, the functional connectivity of the left posterior HIP and angular gyrus increased in most patients with the decreased cognitive ability [15]. Focusing on the HIP, many researchers have shown that even in the absence of plaque during the earliest stages of AD, soluble forms of Aβ could drive neuronal hyperactivity in the HIP of APP/PS1 mice [39], which may confirm the increased GCD in the HIP along with cognitive decline. According to our results, the excitability of neurons in the end stage of AD depends on the accumulation of amyloid plaques. To explain that the HIP of patients with MCI is highly active, while the function of other areas of the DMN is decreased, some researchers proposed the hypothesis that the HIP is disconnected from the DMN [40, 41]. Relatively isolated HIP function is characterized by increased local connectivity and worsening clinical features, which can be seen in our analysis. This may be due to the reduced cortical input in disinhibition-like changes in hippocampal activity [41, 42]. In conclusion, GCD may be more sensitive to neuronal hyperexcitability than to neural dysfunction, revealing the destruction of functional connectivity balance in cognitive disorders.

In this study, the essential role of the cerebellum in cognitive impairment was exhibited in both the VBM and GCD analyses. The cerebellum is considered an organ for coordinating movement; however, recent studies have confirmed the close relationship between the cerebellum and cognitive decline. As shown in several studies using structural and functional MRI, the cerebellum has been confirmed to be involved in cognition, affect [43, 44], and working memory [45], which are significant cognitive decline symptoms in patients with AD and MCI. Furthermore, in a study on the mechanism of the cerebellum in AD, Singh-Bains *et al.* found upregulation of neocerebellar amyloid-β when the number of Tau, ubiquitin, or Purkinje cells did not change, which may be related to significant changes in neurovascular and microglial cells at the early stage of AD [46]. From NCs to patients with AD, we found that the overall trend of GM volumes and functional connectivity decreased, which was consistent with the findings of previous studies [47, 48]. The hypothesis of crossed cerebellar diaschisis (CCD) [49], that is, the remote effect of supratentorial dysfunction in the unilateral hemisphere induces contralateral cerebellar hypometabolism, may explain this phenomenon.

Many studies have confirmed that multimodal neuroimaging is superior to single-mode analysis in predicting the progress and diagnosis of cognitive impairment [50, 51]. In our study, VBM and GCD were voxel-based indices used to assess structural brain volume and functional connectivity, respectively. These may be collaborative imaging markers for clinical applications based on a similar analysis principle. VBM is a typical imaging marker used for tracking disease progression. In the previous VBM analysis, hippocampal volume reduction was used as a biomarker of AD [5-7], which was consistent with our study in which the AUC for the ROC curve was 0.938, with a sensitivity of 93.8% and specificity of 87.5%, for the HIP. However, the reason why the alternation in the hippocampus is not the most sensitive is that it can also be seen in schizophrenia [8] and depression [9, 10]. Through the first analysis of GCD in patients with cognitive decline in our study, a close relationship between cognitive impairment (MMSE and FAQ) and GCD changes in several regions in patients with AD was observed, which indicated that altered GCD in these regions could be utilized as an imaging marker in predicting cognitive decline. MMSE scores were positively correlated with the directed functional connectivity of the MTL subsystem and cerebellar regions, suggesting that functional disconnection is clinically relevant in patients with AD. In contrast, the MMSE scores of patients with AD were negatively correlated with the directed functional connectivity of the DMN midline core system and the MTL subsystem regions, which may validate the above hypothesis of neural compensation and neuronal hyperexcitability. In line with the recognized effect on cognitive decline in the cerebellum [16, 46] and PCUN [14, 52] and the significant differences we computed before, we extracted them as ROIs to perform ROC analyses of MCI and AD prediction, respectively. Using VBM combined with GCD in the cerebellum as a biomarker, we could differentiate between patients with MCI and NCs, reaching an AUC of 0.926, sensitivity of 93.8%, and specificity of 81.2%, which indicated that this biomarker was valuable for early detection of MCI. In terms of predicting the progress of cognitive disorders, the biomarkers that can distinguish between patients with AD and those with MCI are anterior cuneiform combined with VBM and GCD, with an AUC of 0.891, a sensitivity of 81.3%, and specificity of 93.7%, which may be verified in clinical practice. Additionally, the biomarkers in the anterior lobe region combined with VBM and GCD can also distinguish between patients with AD and NCs, with an AUC of 0.938, a sensitivity of 81.3%, and a specificity of 100.0%, which may indicate that this biomarker has the potential to help in AD diagnosis.

Our study had some limitations. First, the number of participants was limited, but we might have the opportunity to recruit more clinical patients in the future. Second, only two main conditions of GCD were analyzed in our study owing to limited experimental equipment. Finally, our research content did not cover gene expression data and EEG analysis. In future research, the effect of GCD combined with EEG will be confirmed, and gene data will be taken into consideration.

## CONCLUSION

To the best of our knowledge, this is the first study to observe changes in VBM and GCD in cognitive decline, which could be the result of a disrupted balance of GM volumes and functional connectivity. These findings could enable us to improve our understanding of AD and MCI pathology, evaluate clinical manifestation, and provide useful neuroimaging markers for the early detection, progression prediction, and diagnosis of AD and MCI.

## Figures and Tables

**Table 1:** Demographic and clinical characteristics of the participants.

**Characteristics**	**AD (n=16)**	**MCI (n=16)**	**NC (n=16)**	***P* value**
Age (years)	76.31 ± 3.98	75.56 ± 6.02	74.69 ± 4.66	0.653
Sex (F/M)	7/9	6/10	10/6	0.338
MMSE	20.75 ± 3.71	27.50 ± 1.79	29.19 ± 1.05	< 0.001
GDS	1.31 ± 0.95	2.44 ± 3.44	0.56 ± 1.03	0.055
CDR	0.97 ± 0.34	0.50 ± 0.26	0.03 ± 0.13	< 0.001
FAQ	17.81 ± 6.6	5.06 ± 5.43	0.06 ± 0.25	< 0.001
NPI-Q	3.75 ± 3.21	3.31 ± 4.22	0.44 ± 0.73	0.008

**Note:** *χ^2^*-test was used in sex, ANOVA in age, MMSE, GDS, CDR, FAQ and NPI-Q. **Abbreviations:** AD, Alzheimer’s disease; MCI, mild cognitive impairment; NC, the normal control; MMSE, Mini-mental State Examination; GDS, Geriatric Depression Scale; CDR, Clinical Dementia Rating Scale; FAQ, Functional Activities Questionnaire; NPI-Q, Neuropsychiatric Inventory Questionnaire.

**Table 2:** VBM among-group comparison in Patients with AD, Patients with MCI, and NCs (*p* < 0.05, GRF corrected).

**Indices**	**Region**	**Brodmann’s Area**	**MNI Coordinates**	***T* value**	**Cluster** **(Voxels)**
ANOVA					
VBM	HIP.L	BA20	(-36,-27,-10)	27.3207	1802
	HIP.R	BA20	(36,-27,-12)	15.7009	1815
	PCUN.L	BA7	(-19,-61,32)	5.2823	91
	PCUN.R	-	(13,-57,31)	7.1891	426
	ACG.L	-	(0,24,27)	6.8790	316
	ACG.R	BA24	(2,25,26)	7.0565	352
	IPL.L	BA40	(-46,-51,49)	8.8743	2100
	IPL.R	BA40	(48,-51,51)	5.2160	82
	Cerebelum_crus1_R	-	(56,-66,-29)	5.3413	62
MCI *vs.* NC					
VBM	HIP.L	BA20	(-37,-17,-19)	-2.9248	28
	MFG.L	BA9	(-29,45,38)	-2.3166	18
	PCUN.R	BA7	(11,-79,56)	-2.5638	19
	ACG.L	BA32	(-37,-37,52)	-3.1472	47
	ANG.L	BA39	(-52,-67,34)	-2.4338	55
	MFG.L	BA9	(-22,32,53)	2.9841	63
	PCUN.L	BA7	(-13,-66,46)	3.5674	16
	PCG.L	-	(-9,-38,17)	2.5892	12
	Cerebelum_crus1_L	-	(-52,-72,-29)	3.5484	614
AD *vs.* NC					
VBM	HIP.L	BA27	(-23,-36,-1)	-5.6276	1923
	HIP.R	BA37	(35,-33,-9)	-5.7248	2019
	MFG.L	BA44	(-48,13,38)	-3.6516	4838
	MFG.R	BA44	(42,13,39)	-6.2246	4393
	PCUN.L	-	(-9,-48,38)	-4.1069	2705
	PCUN.R	-	(7,-44,46)	-4.7299	4742
	PCG.L	BA23	(-9,-52,34)	-3.7718	199
	PCG.R	BA23	(9,-49,32)	-3.7465	105
	TPOsup.L	BA38	(-35,18,-29)	-5.1640	1905
	TPOsup.R	BA38	(45,19,-29)	-3.4944	1463
	Cerebelum_crus1_L	BA19	(-41,-67,-18)	-2.9942	43
	Cerebelum_crus1_R	-	(32,-79,-18)	-2.0795	26
	PCUN.L	BA29	(-5,-42,11)	3.0387	5
	PCG.L	BA29	(-6,-41,12)	2.9175	54
	PCG.R	-	(5,-36,15)	2.9686	54
	Cerebelum_crus1_R	-	(57,-66,-29)	3.2262	224
AD *vs.* MCI					
VBM	HIP.L	BA20	(-27,-10,-12)	-5.7549	1814
	HIP.R	BA36	(28,-7,-25)	-7.9678	1935
	PCUN.L	-	(-12,-43,46)	-4.1613	3925
	PCUN.R	-	(17,-55,33)	-5.0906	4636
	PCG.L	BA23	(-9,-50,34)	-3.2145	370
	PCG.R	-	(5,-54,32)	-2.5907	176
	Cerebelum_crus1_L	-	(-45,-70,-18)	-4.3875	295
	Cerebelum_8_R	-	(21,-42,-57)	2.7028	116

**Table 3:** GCD among-group comparison in Patients with AD, Patients with MCI, and NCs (*p* < 0.05, GRF corrected).

**Indices**	**Region**	**Brodmann’s Area**	**MNI Coordinates**	**T value**	**Cluster** **(Voxels)**
ANOVA					
Inflow	Cerebelum_crus1_L	-	(-24,-64,-37)	7.6918	125
	Cerebelum_8_L	-	(-24,-62,-39)	9.1597	197
Outflow	IPL.L	BA40	(-55,-43,37)	7.0720	43
	Cerebelum_crus1_L	-	(-39,-41,-34)	6.7616	94
MCI *vs.* NC					
Inflow	MFG.L	BA47	(-28,45,9)	-3.5981	189
	ACG.L	BA10	(-6,47,-1)	-3.3719	51
	ACG.R	BA11	(8,33,0)	-3.0331	14
	HIP.L	BA20	(-25,-15,-14)	3.5927	53
	Cerebelum_crus1_L	BA18	(-21,-82,-23)	3.3014	191
	Cerebelum_8_L	-	(-22,-58,-53)	3.5921	171
Outflow	MFG.R	BA9	(22,25,38)	-4.3113	530
	ANG.L	BA22	(-56,-55,25)	3.5306	299
	Cerebelum_crus1_L	BA37	(-46,-57,-22)	5.2767	191
	Cerebelum_8_L	-	(-22,-64,-50)	3.0978	171
AD *vs.* NC					
Inflow	HIP.L	BA37	(-36,-33,-6)	5.0361	72
	PCUN.L	BA29	(-13,-45,10)	3.7396	59
	PCUN.R	-	(7,-59,46)	4.2861	165
Outflow	PCUN.L	-	(-12,-57,-33)	3.6758	268
	PCUN.R	BA7	(6,-63,42)	3.5868	169
	PCG.L	BA23	(-11,-50,30)	2.9622	22
	PCG.R	BA23	(8,-40,30)	2.6875	16
AD *vs.* MCI					
Inflow	Cerebelum_crus1_L	BA18	(-24,-80,-19)	-3.4685	415
	Cerebelum_8_L	-	(-24,-64,-40)	-4.0198	373
	HIP.L	BA27	(-14,-38,9)	-2.9815	25
	PCUN.L	BA23	(-12,-53,28)	3.7315	142
	PCUN.R	BA17	(16,-54,20)	5.1272	318
	PCG.L	BA23	(-6,-53,32)	3.1981	20
	PCG.R	BA23	(10,-39,32)	4.1817	32
Outflow	PCUN.R	BA17	(21,-54,22)	5.2309	336
	PCG.L	-	(-12,-51,30)	4.5910	65
	PCG.R	BA26	(9,-45,24)	2.8261	66

## Data Availability

Owing to the data source of our work, we analyzed the MRI data from ADNI Online Database which contains detailed ethics information of researchers uploaded data respectively. These data are available in the ADNI database (http://adni.loni.usc.edu). The investigators within the ADNI contributed to the design and implementation but did not participate in this analysis.

## LIST OF ABBREVIATIONS

AD: Alzheimer's Disease
Aβ: Amyloid-β Protein
VBM: Volume-based Morphometry
HIP: Hippocampus
NCs: Normal Controls
MCI: Mild Cognitive Impairment
DMN: Default Mode Network
MTL: Medial Temporal Lobe
PCUN: Precuneus
CSF: Cerebrospinal Fluid
