# Supplementary material for: Disrupted Balance of Gray Matter Volume and Directed Functional Connectivity in Mild Cognitive Impairment and Alzheimer’s Disease
Source: Curr Alzheimer Res. 2023 Aug 3;20(3):161–74. doi: 10.2174/1567205020666230602144659 (PMC10514512; doi:10.2174/1567205020666230602144659)
Supplement: Supplementary file 1 [file CAR-20-161_SD1.pdf]

Supplementary Materials

Disrupted Balance of Gray Matter Volume and Directed Functional Connectivity in Mild Cognitive Impairment and Alzheimer's Disease

Yu Xiong[1,#], Chenghui Ye[1,#], Ruxin Sun[1,#], Ying Chen[1], Xiaochun Zhong[1], Jiaqi Zhang[1], Zhanhua Zhong[1], Hongda Chen[2,*] and Min Huang[1,*]

[1]Department of Neurology, the Seventh Affiliated Hospital of Sun Yat-sen University, Shenzhen, Guangdong 518107, China; [2]Department of Traditional Chinese Medicine, the Seventh Affiliated Hospital of Sun Yat-sen University, Shenzhen, Guangdong 518107, China

Table S1. GCD among-group comparison in the AD and NC groups ($p < 0.01$, no correction).

Conditions	Region	Brodmann's Area	Montreal Neurologic Institute Coordinates	T value	Cluster (Voxels)
AD VS NC					
Inflow	MFG.R	BA46	(36,33,40)	-3.5316	12
	TPOsup.L	BA38	(-45,24,-23)	-3.2973	13
	TPOsup.R	BA38	(47,25,-25)	-3.8228	21
	Cerebelum_crus1_R	BA19	(38,-78,-23)	-3.5927	23
Outflow	MFG.L	BA46	(-44,53,9)	-3.3336	8
	MFG.R	BA9	(22,25,41)	-3.6708	13
	PCUN.R	BA5	(9,-48,74)	-3.1110	7
	TPOsup.L	BA38	(-49,20,-23)	-3.4803	8
	TPOsup.R	BA38	(51,25,-21)	-5.3451	35
AD VS MCI					
Inflow	TPOsup.L	BA38	(-44,19,-24)	-4.2939	16
	TPOsup.R	BA38	(48,24,-26)	-3.4157	15
Outflow	TPOsup.L	BA38	(-44,20,-25)	-4.8389	44
	TPOsup.R	-	(48,21,-30)	-3.4201	30
	Cerebelum_crus1_L	-	(-36,-42,-36)	-3.5218	10
	Cerebelum-8_L	-	(-24,-66,-39)	-4.0476	107

1567-2050/23 © 2023 Bentham Science Publishers

Table S2. Classification performances of VBM and GCD metrics ($p < 0.05$).

Conditions	Regions	*P* Value	AUC Value	Sensitivity	Specificity
NC to MCI					
VBM	PCUN.L	0.035	0.719	62.5%	75.0%
	PCG.L	0.016	0.750	75.0%	75.0%
	Cerebelum_crus1_L	0.019	0.742	62.5%	81.2%
Inflow GCD	MFG.L	0.004	0.797	87.5%	62.5%
	ACG.L	0.005	0.789	81.3%	75.0%
	ACG.R	0.006	0.785	93.8%	62.5%
	HIP.L	0.004	0.797	87.5%	68.7%
	Cerebelum_crus1_L	0.007	0.777	56.3%	93.7%
	Cerebelum_8_L	0.005	0.789	81.3%	75.0%
Outflow GCD	MFG.R	0.001	0.852	93.8%	62.5%
	ANG.L	0.003	0.813	75.0%	82.2%
	Cerebelum_crus1_L	0.000	0.906	81.3%	93.7%
	Cerebelum_8_L	0.005	0.789	81.3%	68.7%
Inflow+Outflow GCD	Cerebelum_crus1_L	0.000	0.926	93.8%	81.2%
	Cerebelum_8_L	0.003	0.805	75.0%	75.0%
VBM+Inflow GCD	Cerebelum_crus1_L	0.001	0.855	81.3%	81.2%
VBM+Outflow GCD	Cerebelum_crus1_L	0.000	0.914	81.3%	93.7%
VBM+Inflow +Outflow GCD	Cerebelum_crus1_L	0.000	0.926	93.8%	81.2%
NC to AD					
VBM	HIP.L	0.000	0.938	93.8%	87.5%
	HIP.R	0.000	0.898	81.3%	93.7%
	MFG.L	0.002	0.816	68.8%	93.7%
	MFG.R	0.001	0.836	100.0%	62.5%
	PCUN.R	0.018	0.746	81.3%	62.5%
	PCG.L	0.050	0.703	81.3%	56.2%
	PCG.R	0.050	0.703	75.0%	68.7%
	TPOsup.L	0.007	0.777	75.0%	75.0%
	TPOsup.R	0.097	0.072	43.8%	87.5%
	Cerebelum_crus1_L	0.038	0.715	81.3%	62.5%
	PCUN.L	0.042	0.711	43.8%	100.0%
	PCG.R	0.004	0.801	87.5%	75.0%
	Cerebelum_crus1_R	0.004	0.801	75.0%	87.5%
Inflow GCD	HIP.L	0.000	0.898	87.5%	81.2%

Conditions	Regions	*P* Value	AUC Value	Sensitivity	Specificity
	PCUN.L	0.000	0.898	81.3%	87.5%
	PCUN.R	0.002	0.828	87.5%	68.7%
	IPL.R	0.029	0.727	87.5%	62.5%
Outflow GCD	PCUN.R	0.001	0.836	68.8%	87.5%
	PCG.L	0.004	0.801	93.8%	62.5%
	PCG.R	0.013	0.758	87.5%	68.7%
	IPL.L	0.005	0.789	68.8%	87.5%
Inflow+Outflow GCD	PCUN.R	0.000	0.887	81.3%	87.5%
VBM+Inflow GCD	HIP.L	0.000	0.961	93.8%	87.5%
	PCUN.L	0.000	0.898	87.5%	87.5%
	PCUN.R	0.001	0.855	81.3%	87.5%
VBM+Outflow GCD	PCUN.R	0.001	0.852	81.3%	81.2%
	PCG.L	0.000	0.867	87.5%	75.0%
	PCG.R	0.007	0.777	75.0%	75.0%
VBM+Inflow+ Outflow GCD	PCUN.R	0.000	0.891	81.3%	93.7%
MCI to AD					
VBM	HIP.L	0.001	0.836	81.3%	75.0%
	HIP.R	0.024	0.734	81.3%	56.2%
	PCUN.L	0.038	0.715	81.3%	62.5%
	PCUN.R	0.007	0.781	87.5%	68.7%
	Cerebelum_crus1_L	0.022	0.738	87.5%	62.5%
	Cerebelum_8_L	0.010	0.766	81.3%	75.0%
Inflow GCD	Cerebelum_crus1_L	0.005	0.789	87.5%	62.5%
	Cerebelum_8_L	0.001	0.832	68.8%	87.5%
	HIP.L	0.004	0.801	81.3%	75.0%
	PCUN.L	0.001	0.844	75.0%	93.7%
	PCUN.R	0.000	0.934	87.5%	100.0%
	PCG.L	0.005	0.789	81.3%	62.5%
	PCG.R	0.001	0.848	56.3%	100.0%
Outflow GCD	PCUN.R	0.000	0.895	93.8%	75.0%
	PCG.L	0.000	0.918	93.8%	81.2%
	PCG.R	0.006	0.785	68.8%	87.5%
Inflow+Outflow GCD	PCUN.R	0.000	0.938	81.3%	100.0%
	PCG.L	0.000	0.930	93.8%	81.2%
	PCG.R	0.000	0.891	93.8%	81.2%
VBM+Inflow GCD	HIP.L	0.000	0.887	81.3%	81.2%

Conditions	Regions	*P* Value	AUC Value	Sensitivity	Specificity
	PCUN.L	0.001	0.848	68.8%	87.5%
	PCUN.R	0.000	0.934	87.5%	100.0%
	Cerebelum_crus1_L	0.002	0.828	68.8%	87.5%
	Cerebelum_8_L	0.000	0.914	81.3%	87.5%
VBM+Outflow GCD	PCUN.R	0.000	0.918	93.8%	81.2%
VBM+Inflow +Outflow GCD	PCUN.R	0.000	0.938	81.3%	100.0%
